# Polymer-II-VI Nanocrystals Blends: Basic Physics and Device Applications to Lasers and LEDs

**DOI:** 10.3390/nano9071036

**Published:** 2019-07-19

**Authors:** Marco Anni

**Affiliations:** Dipartimento di Matematica e Fisica “Ennio De Giorgi”, Università del Salento, Via per Arnesano, 73100 Lecce, Italy; marco.anni@unisalento.it; Tel.: +39-0832-297540

**Keywords:** conjugated molecules, II-VI semiconductors, NCs, hybrid films, laser, light emitting diodes, solar cells, perovskites

## Abstract

Hybrid thin films that combine organic conjugated molecules and semiconductors nanocrystals (NCs) have been deeply investigated in the previous years, due to their capability to provide an extremely broad tuning of their electronic and optical properties. In this paper we review the main aspects of the basic physics of the organic–inorganic interaction and the actual state of the art of lasers and light emitting diodes based on hybrid active materials.

## 1. Introduction

The need for active materials for photonic, optoelectronic and electronic devices with improved performances strongly stimulates the research on innovative materials with semiconducting properties, able to provide advantages with respect to standard inorganic semiconductors in terms of realization costs, active properties engineering and absolute performances. In this very broad field, particular attention has been devoted in the last two decades to conjugated organic molecules, both oligomers and polymers and to semiconductor nanocrystals (NCs).

Conjugated molecules are characterized by a combination of the processability and low costs typical of plastic materials with active properties, like charge mobility, luminescence and optical gain, typical of semiconductors.

**Figure 1:**
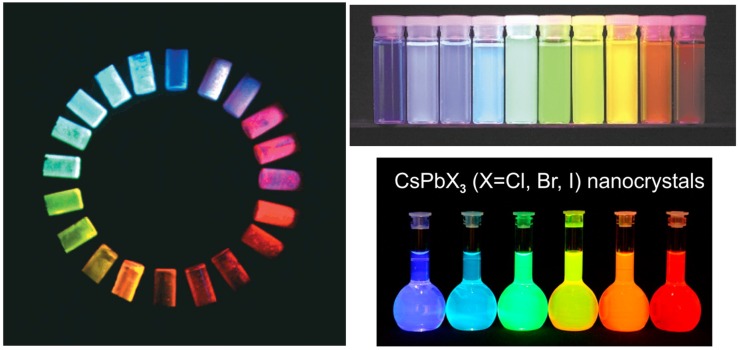
(**left**) Photoluminescence color tunability obtained by functionalized dioxide-oligothiophenes with different conjugation length. Reprinted with permission from [1]. American Chemical Society, 2000. (**top right**) Photoluminescence color tunability in II–VI quantum dots with different sizes. Adapted with permission from [31]. Springer Nature Publishing, 2001. (**bottom right**) Full emission color tunability of fully inorganic CsPbX${}_{3}$ NCs with different chemical composition. Reproduced with permission from [32]. American Chemical Society, 2015.

Moreover, they show a strong dependence of the electronic properties on the molecular chemical structure, that allows us to widely tune their properties by properly engineer the molecular backbone. For example light emission fully tunable in the visible–near infrared (VIS-NIR) range has been demonstrated in many classes of molecules by controlling the electron wavefunction delocalization along the molecule acting on the molecule chemical structure [1] (see Figure 1 left).

A proper molecule functionalization can be also introduced to make the molecules soluble in common organic solvents, thus allowing the easy deposition of active layers by spin coating [2], drop casting [3], layer by layer deposition [4], ink jet printing [5], spray coating [6] and pulsed laser deposition [7,8].

To date, several demonstrators of organic optoelectronic and photonic devices have been reported like light emitting diodes [9], solar cells [10,11,12] and lasers [13].

Concerning NCs, after the first demonstration of simple synthesis of spherical CdSe, CdS and CdTe NCs [14], an enormous interest has been devoted to the development of new synthesis processes able to realize colloidal NCs with controlled size, shape, electronic and optical properties. A peculiar property of semiconductor NCs is the possibility to get full emission color tunability by realizing NCs smaller than the exciton Bohr radius, thus changing the electronic levels thanks to quantum size effect (see Figure 1 top right). The photoluminescence quantum yield (PLQY) of NCs is strongly limited by the presence of a high density of superficial defects. An elegant solution of this problem is the overgrowth of the core NCs with a thin shell of a wider gap semiconductor, resulting in an efficient surface passivation [15,16,17]. Further works demonstrated the possibility to finely control not only the chemical compositions and the size of the NCs, but also their shape in order to realize elongated quantum rods [18], tetrapods [19], teardrops, branched tetrapods [20] and dot in rods NCs [21]. Further details on the synthesis, characterization and applications of II-VI colloidal NCs can be found in several excellent recent review papers [22,23,24,25,26,27,28,29,30].

The wide engineering possibilities of both conjugated molecules and of inorganic NCs also stimulated the research on hybrid organic–inorganic composite materials, aiming to combine the best of the two worlds in order to overcome the individual limitations of the two families. For example, hybrid materials can exploit the good film forming properties of many organic molecules in order to improve the film forming properties of inorganic NCs, or they can show improved charge transport properties with respect to fully organic active layers, thus exploiting the typically much higher charge mobilities of inorganic materials.

In this paper we will review the main aspects related to light matter interaction in hybrid organic–inorganic polymer-NC films. In particular we will describe how optical spectroscopy experiments allow to investigate the organic-NC interaction and how the physical properties of hybrid films have been exploited for the realization of active materials for lasers and light emitting diodes. Finally we will present the possible development perspectives of hybrid active materials.

## 2. Physical Aspects of Polymer: NCs Photophysics

### 2.1. Basic Aspects of Polymer: NC Interaction

In order to understand the electronic and optical properties that can be obtained by combining a conjugated molecule with a NC it is absolutely mandatory to have a deep understanding of the interactions between the two systems that can take place in a hybrid film. This aspect is fundamental to allow the development of electronic, optoelectronic and photonic hybrid devices but, on the other side, it is also extremely interesting for basic science experiments.

The first complete experiment on the photophysics of hybrid organic–inorganic films has been performed by N. Greenham et al. [33] that realized hybrid active films by blending the red emitting polymer poly(2-methoxy,5-(2’-ethyl)-hexyloxy-p-phenylenevinylene (MEH-PPV) with CdSe and CdS NCs, that have an energy gap smaller and larger than the MeH-PPV one, respectively. The optical properties of the films were investigated by measuring the polymer photoluminescence (PL) intensity and the film photoconductivity dependence on the NC content of the blend. The main result of the experiment was that the MeH-PPV PL is not affected by the presence of CdS NCs when their surface is covered by tri-octylphosphineoxide (TOPO) surfactant, while a clear PL quenching, increasing with the NCs content, was observed if the surfactants were removed. A different behavior was instead observed in MeH-PPV:CdSe films, with a clear PL quenching even in TOPO covered NCs and a stronger quenching in absence of surfactants. The results on MeH-PPV:CdS with TOPO were ascribed to the lack of energy and charge transfer from the polymer to the CdS NCs due to the higher energy gap of CdS with respect to MeH-PPV, preventing energy transfer, and to the presence of the ligand, preventing electron transfer from MeH-PPV to CdS NCs. The ligand removal allowed the excitons photogenerated in MeH-PPV to reach the NCs surface, and to be then quenched due to electron transfer toward the NC. Concerning MeH-PPV:CdSe films the MeH-PPV PL quenching in films with TOPO covered NCs was ascribed to Förster resonant energy transfer (FRET) from MeH-PPV to the NCs, and the further quenching when the TOPO was removed to the addition of charge transfer to the energy transfer. Overall, this experiment demonstrated that the organic and the inorganic components can interact by FRET and/or electron transfer depending on the relative values of the energy gap and on the surface functionalization of the NCs. The attribution of part of the quenching to electron transfer toward the NCs was also validated by photoconductivity experiments, evidencing an increase photoconductivity with the NCs content and also proposing for the first time the conjugate polymer- NCs blends as potential active layers in photovoltaic devices.

The same group also demonstrated [34] that the polymer to NC electron transfer is strongly dependent not only on the presence of TOPO on the NC surface, but also on the kind of alkyl chains on the polymer backbone. In particular, even if energetically favored, charge transfer does not take place from a PPV derivative containing two dihexyloxy chains on both sides of the polymer chain, preventing NCs from approaching sufficiently close to the conjugated backbone to allow charge transfer to occur.

**Figure 2:**
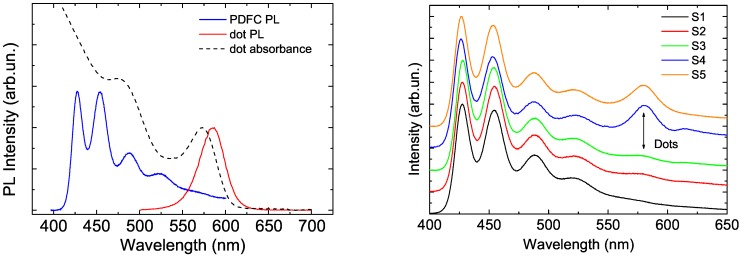
(**left**) Photoluminescence and absorption spectra of the CdSe/ZnS quantum dot, and PL spectrum of poly[(9,9-dihexylfluorenyl-2,7-diyl)-alt-co-(9,ethyl-3,6-carbazole)] (PDFC). (**right**) PL spectra of the PDFC: CdSe/ZnS blends for increasing nanocrystal (NC) content from bottom to top. The arrow indicates the dots emission. Adapted with the permission from [35]. AIP Publishing, 2004.

**Figure 3:**
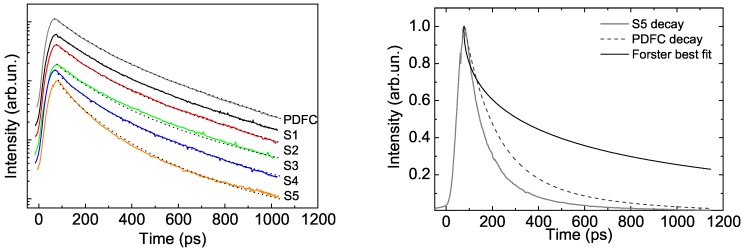
(**left**) Relaxation dynamics of the PDFC: CdSe/ZnS blends for increasing NC content from top to bottom. The dotted lines are the best fit curves. (**right**) Different contributions to the PL relaxation of the PDFC: CdSe/ZnS blend with a CdSe/ZnS content of 1.1%. Adapted with the permission from [36]. AIP Publishing, 2006.

The details of FRET from a conjugated polymer to a NC was later investigated by time resolved photoluminescence spectroscopy by Anni et al. [35] in thin films of a blend between a blue-emitting polymer, namely poly[(9,9-dihexylfluorenyl-2,7-diyl)-alt-co-(9,ethyl-3,6-carbazole)] (PDFC), and colloidal CdSe/ZnS core–shell quantum dots. In this case the NCs surface was covered by TOPO as ligand, thus preventing electron transfer. As FRET is basically related to a resonant energy transfer between an excited dipole, acting as energy donor, and an unexcited dipole, acting as energy acceptor, the first condition to be fulfilled is a good spectral overlap between the donor photoluminescence range and the acceptor absorption one. This condition was clearly realized between PDFC and CdSe/ZnS quantum dots (see Figure 2 left). The PL spectra of the hybrid films showed the superposition of the polymer PL and of the NCs PL, whose contribution increases as the NCs content in the blend was increased (see Figure 2 right). In order to investigate the kind of interactions between the blend components PL time resolved measurement were performed evidencing that the polymer PL relaxation becomes progressively faster as the NC content increased (see Figure 3 left). This result evidences that the presence of the NCs in the film introduced a further decay channel for the photogenerated excitons in the polymer. The variation of the relaxation dynamics and its dependence on the NCs content was found in excellent agreement with the predictions of a model including polymer to NC FRET, for a best fit FRET radius of about 48 Angstrom, in excellent agreement with the value of 56 Angstrom determined from the spectral overlap. The FRET contribution to the polymer relaxation was also found to be faster in the first 100 ps after the photoexcitation, and then to progressively slow down (see Figure 3 right). This behavior suggests that the excitons in the polymer have a negligible diffusion after the photoexcitation, thus the FRET is more likely immediately after the excitation pulse, while the FRET rate progressively decreases in time due to the progressive increase of the average donor–acceptor distance.

A further confirm that the polymer NCs interaction is mainly related to FRET was obtained three years later in poly[9.9-bis(2-ethylhexyl)fluorene-2.7-diyl](PF2/6) and core–shell CdSe/ZnS QDs [37], adding the evidence that the PL from the NCs in the blend has a slow rise time of 63 ps, in excellent agreement with the FRET time constant of 68 ps determined from the analysis of the PF2/6 relaxation dynamics and confirming that the FRET basically takes place within the first 200 ps after the photoexcitation.

Moreover the same group demonstrated that exciton energy migration within the polymer is fundamental to have an efficient energy transfer toward the NCs, as evidenced by the strong energy transfer efficiency increase from cryogenic temperatures to room temperature [38].

These initial works allowed us to fix several important points on the possible processes that can take place in polymer-NC blends also allowing to determine possible strategies to increase the energy transfer efficiency, in order to develop hybrid active materials with engineered light emission properties, or the charge transfer efficiency, in order to develop active materials for photovoltaic devices. In particular the evidence of interaction dependence on the surface functionalization of the NCs stimulated further systematic investigations, like the study of the ligand dependence of charge and energy transfer in blends of MeH-PPV and CdSe quantum dots with TOPO or oleic acid (OA) ligand [39]. This experiment allowed us to observe that the ligand engineering can allow to switch between a system in which the dominant polymer NC is FRET (when TOPO is used) to a system in which the main interaction is charge transfer (when OA is used).

The role of the chemical nature and of the thickness of the NCs shell was also investigated [40] in blend films of 1,3,5-tris(N-phenylbenzimidazol-2,yl) benzene (TPBI) and CdSe quantum dots with CdS shell of two different thicknesses (2 monolayers (ML) and 6 ML), ZnS shell (3 ML), and thick CdS/ZnCdS/ZnS multishell (7 ML). The authors demonstrated that the main interaction process is FRET, whose rate was found to decrease as the CdS shell thickness increases, due to the increase of the average donor–acceptor distance. Moreover an increased FRET rate was found when ZnS shell was used, evidencing that also the position of the shell energy levels is important. Rather interestingly the FRET rate between TPBI and quantum dots with CdS/ZnCdS/ZnS multishell was the same of the one between TPBI and quantum dots with thinner ZnS shell, suggesting that the role of the shell energy levels is stronger than the shell thickness one.

**Figure 4:**
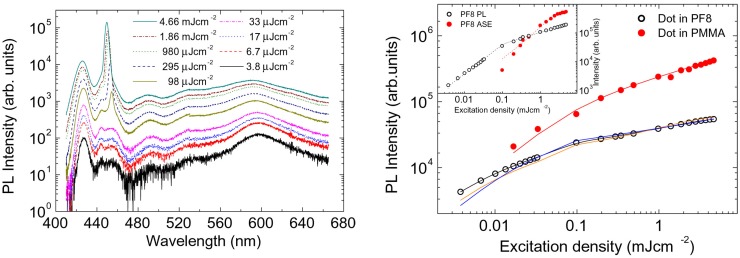
(**left**) Excitation density dependence of the PL spectra of the PF8-NC thin film. (**right**) Integrated PL intensity of the NC emission as a function of the excitation density for the PF8-NC film (empty black dots) and the PMMA-NC one (full red dots). Inset: integrated intensity of the PF8 PL (empty black dots) and amplified spontaneous emission (ASE) (full red dots) in the PF8-NC film as a function of the excitation density. All the lines are the best fit curves. Reprinted with permission from [41]. American Chemical Society, 2010.

We observe that in all the previous experiments, the first effect of the polymer-NC interaction in the hybrid film is the presence of PL quenching of the organic component. However this effect is not by itself enough to understand the kind of interaction taking place as a PL quenching can be caused by exciton transfer toward the NCs, but also by electron transfer, resulting in exciton quenching in the organic. A possible way to separate the two effects is to look at the NC PL intensity, as a NC PL enhancement should be expected only in case of energy transfer. Anyway even an organic PL quenching taking place together with a NC PL enhancement cannot be only due to FRET, as a radiative transfer (thus emission of the organic followed by reabsorption by the NC) also leads to the same effect. For these reasons all the experiments aiming to understand in which way the organic and the inorganic component interacts typically combine PL spectroscopy with other techniques, like photoconductivity, time resolved PL or photoinduced absorption. A possible alternative approach to separate the different NCs excitation processes in active hybrid blends, fully based on PL measurements, has been also demonstrated in hybrid poly(9,9-dioctylfluorene)-CdSe/ZnS NC (PF8-NC) films [41]. In this experiment the active blend PL spectra were compared with the ones of an inert polymer (polymethyl methacrylate, PMMA) PMMA-NC blend, with the same NC content. The PL spectra of the PF8-NC blend show, at low excitation density, the superposition of the PF8 PL (with the 0-0 line at about 428 nm, followed by three vibronic replicas at about 450, 490, and 530 nm, respectively) and a clear NC PL peak at about 600 nm (see Figure 4 left). As the excitation density increased a clear PF8 amplified spontaneous emission band was observed above 100 μJ cm${}^{-2}$, progressively dominating the blend spectrum. Moreover a clear reduction of the NCs relative contribution to the blend PL was observed.

**Figure 5:**
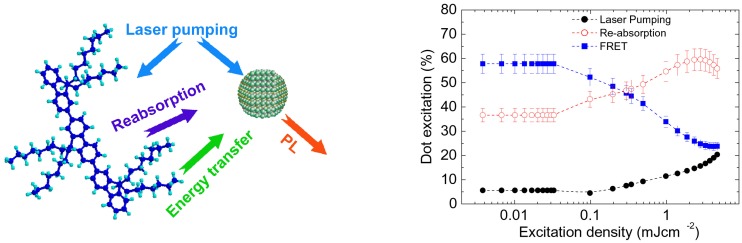
(**left**) Pictorial view of the PF8 and NCs excitation processes leading to NC PL emission. (**right**) Relative weight of the NCs pumping processes as a function of the excitation density. Reprinted with permission from [41]. American Chemical Society, 2010.

In order to understand the processes leading to NCs excitation in the blend the attention was focused on the excitation density dependence of the integrated NCs PL intensity. In particular it was observed that the excitation density dependence of the NCs integrated PL intensity in the PMMA-NC blend showed an initial linear increase up to an excitation density of about 150 μJ cm${}^{-2}$, followed by a sublinear increase (see Figure 4 right). This behavior evidences that single exciton relaxation processes are present up to 150 μJ cm${}^{-2}$, while the sublinear increase at higher excitation density is the typical signature of non linear nonradiative processes, like Auger scattering [42]. Concerning the NCs PL in the PF8-NCs blend a stronger sublinearity was observed at all the investigated excitation densities (see Figure 4 right), evidencing a higher NCs average population. Starting from the rate equation for the NCs excited state population, the exciton lifetime and the Auger constant of the NCs were obtained from the data of the PMMA-NCs blend, and the differences observed in the PF8-NC blend analyzed including as NCs excitation processes direct pump laser absorption, energy transfer from the polymer and polymer PL reabsorption (see Figure 5 left). This procedure allowed us to determine the NCs pumping rate of all the processes and thus to quantify their relative importance. In particular it was demonstrated (see Figure 5 right) that at low excitation density about 95% of the NCs are excited by the polymer (about 58% by FRET and about 37% by PF8 PL absorption). Above the amplified spontaneous emission (ASE) threshold a progressive decrease of the relative FRET importance was observed, and a complementary increase of the reabsorption. This result was ascribed by the PF8 exciton lifetime decrease induced by ASE, leading to a faster PF8 relaxation that decreases the FRET probability and to a strong increase of the PF8 emission, leading to a reabsorption importance increase.

Even if experiments on blend films allowed us to observe several interesting features related to the organic–inorganic interactions that can be directly exploited for the realization of active layers in photonic and electronic devices, sample’s local non uniformities leading due to non uniform organic–inorganic mixing, caused by polymer and NCs aggregation, cannot in general be excluded in blend films and they can strongly affect the energy and charge transfer efficiency [43]. An important step in the possibility to control the properties of hybrid materials came then from the realization of organic–inorganic nanocomposites in which the distance between the components is carefully controlled.

A physical approach to get this result is the realization of multilayers, in which alternated organic and NCs thin films are sequentially deposited. This approach was exploited, for example, in order to investigate the organic inorganic interactions between a phosphorescent organic film, made of Ir(ppy)3d oped into 4,4’-*N*,*N*’-dicarbazole-byphenyl (CBP), and a monolayer of CdSe/ZnS QDs deposited on the film surface [44]. The experiment allowed us to observe a QDs PL enhancement due to energy transfer from the organic film, with a Förster Radius of 4.1 nm, thus providing the first evidence of energy transfer to a QD from a triplet state of an organic donor.

A layer by layer (LbL) alternated organic–inorganic film was also realized by growing a multilayer made of the water-soluble conjugated polymer poly(9,9-bis(3’-[(*N*,Ndimethyl)-*N*- ethylammonium]-propyl)-2,7-fluorene-alt-1,4-phenylene]dibromide (PDFD) and CdTe NCs capped with thioglycolic acid molecules, exploiting of the water solubility of positively charged PDFD to use it as a counterpart for negatively charged CdTe NCs in the LbL assembly [45]. The investigation of the temperature dependence of the PL spectra of the samples allowed us to demonstrate that the energy transfer efficiency in hybrid organic/inorganic nanocomposites of conjugated polymers and semiconductor NCs is the result of the competition between the energy transfer process from the polymer to NCs and the nonradiative recombination in the polymer. Moreover is was shown that in the LbL assembled nanocomposites the layers of semiconductor NCs behaves both as very efficient energy acceptors and as insulating layers effectively suppressing nonradiative recombination in conjugated polymer layers via reduction of the exciton diffusion toward non radiative defects.

A high control of the organic–inorganic distance can also be obtained by chemical approaches, in order to realize chemically bonded nanocomposites, that also allow the control of the number of molecules interacting with the NC [46], and to predict the efficiency of energy transfer [47].

A very elegant experiment in this direction was performed by the J. Feldmann’s group [48] that realized nanocomposites made by CdTe quantum dots and a water soluble polyfluorene copolymer. The NC polymer assembly was obtained thanks to negatively charged ligands on the NCs surface and positively charged chains on the polymer backbone. These systems were then characterized by a controlled distance, while the role of aggregations was excluded by working in solution. The interesting conclusion of the experiments was that the nanocomposites showed a quenched polymer PL and an enhanced NC PL, clearly evidencing the presence of energy transfer from the polymer to the NCs. However a quantitative comparison between the energy transfer efficiency determined from the polymer intensity quenching (estimated in 94%) and from the NCs intensity enhancement (72%) clearly demonstrated that not all the exciton quenched in the polymer are transferred to the NCs. A detailed comparison between the polymer and the NCs PL relaxation dynamics variations in the composite finally allowed us to demonstrate that the polymer PL is mainly quenched due to FRET to the NCs, but also charge transfer takes place, resulting in a polymer PL quenching but not in a NC PL enhancement. This non unique behavior was ascribed to the dependence of the FRET on the relative orientation of the donor dipole and the acceptor one, that prevents FRET in absence of a proper relative orientation, thus allowing charge transfer.

Similar evidences of efficient organic–inorganic interaction were also obtained in oligothiophene-CdSe quantum rods nanocomposites [49], showing that the attachment of the oligothiophenes to the CdSe nanoparticles results in little change to the electronic structure of the oligothiophenes, but in a strong quenching of the oligothiophene PL, ascribed to either an electron or energy transfer mechanism, while no appreciable quenching of the quantum rods evidenced the lack of hole transfer from the rod to the oligothiophene.

The importance of the control of the donor–acceptor interaction was also clearly evidenced by the strongly increased energy transfer from oligophenylene-vinylene molecules to CdSe quantum dots in nanocomposites with respect to blend films [50].

**Figure 6:**
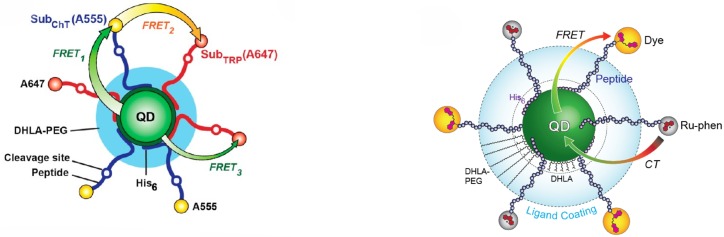
(**left**) Design of the concentric FRET relay and three possible energy transfer pathways between the QD, A555, and A647. The dye-labeled peptide substrates (Sub) are assembled to poly(ethylene glycol) ligand (DHLA-PEG)-coated CdSe/ZnS QDsviapolyhistidine (His6) tails. Reprinted with permission from [58]. American Chemical Society, 2012. (**right**) Schematic illustration of the combined CT and Förster resonant energy transfer (FRET) system constructed around a central QD using self-assembled polyhistidine-terminated peptides. The peptides are labeled at their distal termini with either a ruthenium(II) phenanthroline complex (Ru-phen) or afluorescent dye (A555, Cy3, A594). The QD is coated with either DHLA-PEG or DHLA ligands. Reprinted with permission from [54]. American Chemical Society, 2017.

The possibility to finely control the length and the chemical nature of the spacer between the organic and the inorganic part of the nanocomposite allows many degrees of freedom for the realization of nanocomposites with predictable type and strength of the interaction between the components. The modulation of this interaction can be used to obtain sensing properties of the nanocomposites, as widely demonstrated in literature in the last years [51,52,53]. In particular it has been demonstrated that NC-organic nanocomposites with multiple functionality can be realized by integrating a central NCs with multiple energy/charge transfer channels. Several approaches have been reported to date, like systems in which the NC acts both as energy acceptor from a first organic molecule and as energy donor toward a second molecule by using either external excitation [54,55] or bioluminescence [56,57] as excitation process of the first donor, or systems in which the same NC can act as energy donor of two different organic acceptors [58] (see Figure 6 left).

The possibility to combine energy and charge transfer toward two different organic acceptors (see Figure 6 right) has been also recently demonstrated as a powerful way to obtain multiplexed sensing of protease activity [54].

### 2.2. Basic Aspects of Light Amplification in NCs

The development of colloidal NCs to be used as active materials in light amplifiers and lasers received large attention in the last two decades, after the first demonstration of optical gain in NCs thin films by V. Klimov et al. [59] in CdSe quantum dots films. This first breakthrough evidenced on one side that colloidal NCs can show high optical gain but, on the other side, that the small size regime necessary to fully exploit the emission tunability of colloidal NCs also leads to detrimental effects on the gain properties. In particular the small Stoke’s shift between the NCs absorption and photoluminescence makes these NCs a quasi 2 levels system thus requiring, in order to get population inversion, the creation of at least two excitons in at least part of the NCs. The negative consequence of the combination of biexciton creation and strong exciton confinement is that efficient non radiative Auger scattering quickly reduces the population inversion, thus switching off the gain on the few picoseconds time scale [42]. This intrinsic limitation of NCs for lasers applications stimulated the development of many approaches to limit the Auger induced excitons non radiative relaxation.

The physical approaches to reach this result include the use of ultrashort pumping pulses, that allow to almost instantaneously invert the population in times shorter than the Auger lifetime. This approach was used to observe stimulated emission in many colloidal NCs in basic science experiments but, on an applicative perspective, it is not compatible with the target of developing low cost optically pumped NCs lasers, due to the too high costs of amplified femtoseconds lasers.

A second possible strategy exploits the dependence of the population inversion on the NC density in the film, that allows to increase it, without increasing the Auger rate, by developing high density compact films [59].

Several chemical approaches have been also demonstrated, in order to reduce the Auger scattering by designing novel nanostructures with engineered electron and hole wave-function localization.

A first possibility is to reduce the wavefunction confinement by realizing elongated NCs (quantum rods) [18,20]. The transition from 0D to 1D NCs allows to strongly decrease the Auger rate, thus strongly increasing the gain lifetime [60]. Similar results can be obtained by realizing dot-in-rod NCs, in which a spherical quantum dots serve as core embedded in a rod-shaped shell [21].

Another possible approach is to realized the so called type-II core/shell NCs [61], like CdSe/ZnTe, in which the shell layer has a higher energy gap than the core and it has both the valence and conduction bands lower, or higher, than in the core. As a consequence one carrier is mainly confined in the core, while the other is mainly confined in the shell, thus reducing the wavefunction overlap and thus the scattering probability.

A third way to control and reduce the Auger scattering rate is the realization of core/shell NCs, in which a gradual transition from the core material to the shell one is present. The first demonstration of Auger dependence on the core/shell interface was obtained in CdSe/CdS core/shell QDs in which an intermediate CdSe${}_{x}$S${}_{1-x}$ alloy layer is present [62]. This experiment allowed us to observe a negligible role of the graded shell on the single exciton lifetime, but an appreciable Auger reduction, proposing core/shell surface engineering as a valuable degree of freedom for the control of the NCs properties. The effects of the shell presence and of the core/shell interface sharpness was investigated by comparing the ASE properties of CdZnS core quantum dots, with the ones of CdZnS/ZnS core/shell with both sharp shell (SS) and alloyed shell (AS) [63]. ASE at about 470 nm with a threshold of about 260 μJ cm${}^{-2}$ was observed in the core QDS, with a clear reduction down to 160 μJ cm${}^{-2}$ in the SS core/shell sample and to 55 μJ cm${}^{-2}$ in the AS core/shell.

All these approaches allowed us to obtain remarkable improvements of the optical gain properties, recently allowing the demonstration of continuous wave lasing at room temperature in colloidal nanoplatelets [64,65].

## 3. Hybrid Polymer: NCs Devices

### 3.1. Lasers

Several optically pumped lasing devices based on NCs have been reported to date, exploiting different active materials, laser resonator geometries and realization procedures [23]. In order to ensure optimized lasing threshold it is mandatory to realize active materials with good optical quality, ensuring low propagation losses along the cavity. Moreover laser devices needs to show good operational stability, and the active layer must have precise physical properties, like thermoplasticity, to allow the use of low cost fabrication techniques, like soft-lithography ones. In order to address these two issues composite organic–inorganic materials have been proposed in order to overcome the intrinsic limits of NCs. In particular, it has been demonstrated that the lack of operational stability in NC based lasers is related to degradation caused by the interaction of the ligands with oxygen and water [66] and the inclusion of the NCs in a matrix has been demonstrated as valuable strategy to improve the operational stability. The first exploited approach reported in literature is based on the inclusion of the NCs in sol gel titania or silica matrix. In particular good lasing stability was reported for CdSe/CdZnS embedded in a titania matrix [67]. It was later demonstrated that titania shows strong degradation, evidenced by film cracking, when exposed to moisture and water and that much better stability can be obtained by exploiting silica matrices [68].

In the following we will describe the properties of different optically pumped NC lasers, trying to provide a general overview of the variety of spectral properties obtained to date, and evidencing the results made possible by the development of hybrid organic–inorganic materials.

**Figure 7:**
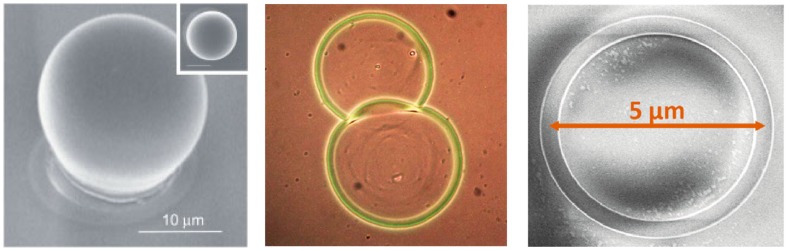
**Left**: SEM image of a 20 μm diameter silica microsphere coated by a CdSe/CdZnS titania film. Reproduced with permission from [67]. WILEY-VCH Verlag GmbH & Co. KGaA, 2005. **Center**: Microscope image of a coffee ring microresonator. Reproduced with permission from [69]. The Royal Society of Chemistry, 2010. **Right**: SEM image of a template stripped microring. Reprinted from [73]. American Chemical Society, 2018.

A very diffused cavity type in NCs lasers realized is the whispering gallery modes (WGM) one, in which the active material is in a micrometric resonant cavity with spherical or cylindrical symmetry, like spheres, disks, rings, and toroids, embedded in a medium with lower refractive index (see Figure 7). The light propagating in the cavity suffers total internal reflection at the cavity edge, and resonant modes are formed at wavelengths allowing constructive interference for propagation around the cavity. These lasers do not require advanced lithographic techniques, as the cavity size is typically on the several microns/tens of microns scale, and are characterized by multimodal emission. The first clear demonstration of WGM lasing from NCs was obtained exploiting silica microsphere as resonators, covered by an active CdSe/CdZnS titania composite [68] (see Figure 7 left). The NCs coated microspheres showed multimode lasing emission in the red region (around 660 nm) with cavity Q factor up to 1000.

More recently several groups obtained laser WGM microcavities by exploiting the coffee-ring effect, thus by simply depositing a drop of the solution containing the NCs, and creating a circular microdisk thanks to the accumulation of the active material on the drop border (see Figure 7 center) [69]. The first demonstrator of this kind of cavities [69] had cavity length between 23 μm and 80 μm and exploited CdSe/CdS Quantum Rods (QRs) as active materials. A cavity Q factor close to 1000, thus comparable with the microspheres one, was demonstrated, allowing to reach a lasing threshold of 3.0 mJ cm${}^{-2}$ under femtosecond excitation.

A strongly reduced threshold was obtained in CdSe/CdS core/shell dot/rod nanorods (NRs) [70] in cavities deposited by capillary jet deposition of picoliter solution drops, allowing to generate in controllable way microring cavities with diameter in the hundred of micrometers scale and width in the 5–15 μm range. The cavities showed quasi single mode lasing from the CdSe core (at 610 nm) or, for the first time, also from the CdS shell (at 495.5 nm) depending on the rod length, with a very low threshold of 0.2 mJ cm${}^{-2}$ under femtosecond excitation. Even lower lasing threshold (15 μJ cm${}^{-2}$) was demonstrated in water soluble core–shell CdSe/CdS quantum dot in rods (DiR) coffee-ring microrings [71], further decreased down to 2 μJ cm${}^{-2}$ in microrings with CdSe/CdS QDs as active material [72], with a remarkable Q factor of 2000, evidencing the high cavity quality that can be obtained with a so simple approach. Quasi toroid resonators with Q factor above 2000 have been also demonstrated by ink-jet printing, leading to multimode lasing around 460 nm with a threshold of 6.7 mJ cm${}^{-2}$ [63].

**Figure 8:**
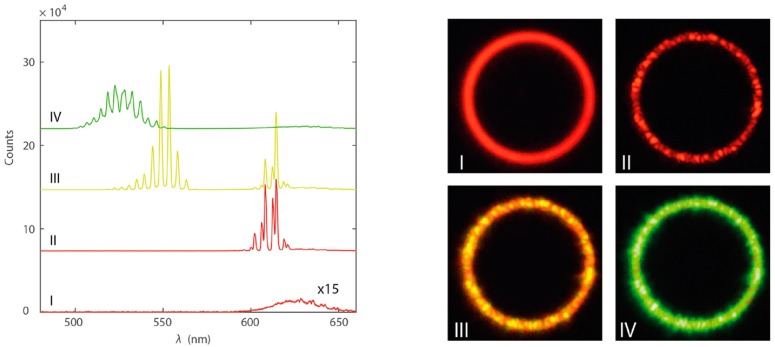
(**left**) Emission spectra of the microring lasers as a function of the excitation density, evidencing the emission color variation. (**right**) True-color photographs of a 4 μm radius QD ring laser excited in the power regimes, I–IV. Adapted from [73]. American Chemical Society, 2018.

Despite the very easy fabrication procedure, that makes coffee ring micro-disks good templates for lasing characterization, the lack of reproducibility of the emission spectra from different devices strongly limits the applicative perspectives of these systems. A noteworthy demonstration of the quality of WGM microring lasing that can be obtained from colloidal NCs, in well controlled lithographycally fabricated devices, and of the emission properties engineering possibilities in colloidal NCs has been recently obtained by le Feber et al. [73]. High quality and highly controlled microrings with variable radius from 0.5 μm to 7 μm (see Figure 7 right), with a quality factor up to 2443, were realized and low threshold lasing was obtained from CdSe/CdS/ZnS core/shell/shell quantum dots. In particular the authors demonstrated the presence of lasing around 610 nm due to emission from the CdSe core with a threshold of 22 μJ cm${}^{-2}$ followed, as the excitation density increases, by red lasing saturation and by the emergence of lasing in the green above 110 μJ cm${}^{-2}$, ascribed to emission from the CdS shell (see Figure 8 left). The possibility to have lasing both from the core and from the shell, together with their individually different excitation density dependence allowed the lasing color tuning from the red to the green (see Figure 8 right).

**Figure 9:**
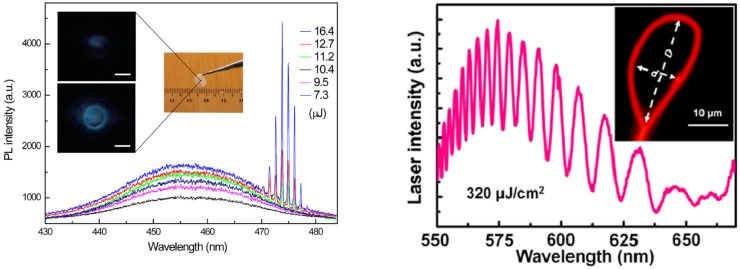
(**left**) Pump power dependent micro PL spectra from a single microbubble with diameter of about 34 μm. The inset shows a photograph of the free-standing CQD/PMMA nanocomposite film and the PL images of the microbubble below (upper one) and above (bottom one) threshold (scale bar 20 μm). Reproduced with permission from [74]. American Chemical Society, 2017. (**right**) multimode laser emission spectrum of a ring resonator at an optical pump of 320 μJ cm${}^{-2}$. Inset: dark-field PL image of the ring resonator with circumference of 70 μm formed by a 500 nm diameter polymer nanowire with D = 26 μm and d = 11 μm. Adapted from [75]. American Chemical Society, 2014.

An innovative type of WGM lasers was also recently demonstrated exploiting a hybrid polymer-NC active system, made by a composite film of PMMA and CdZnS/ZnS QDs [74]. After the drop casting of a toluene solution, the authors observed the spontaneous formation of microbubbles during the solvent evaporation with dimensions depending on the QDs content of the blend and resulting in the formation of hollow domes. These structures showed lasing modes with a Q factor above 2000 (see Figure 9 left) and very high stability, as evidenced by the lack of threshold increase for 12 months of storage in dark, while a one order of magnitude increase of the ASE threshold of a QDs close packed film was observed after 3 months.

A good example of the design flexibility that can be obtained by combining polymer and NCs is provided by the demonstration of WGM lasing from microrings made by bended CdSe–ZnS core–shell quantum dot (QD) doped polyvinylpyrrolidone (PVP) single nanowires [75]. The nanowires were realized by a direct drawing method and show a length on the scale of millimiters, with a diameter between 300 nm and 900 nm. The nanowire bending allowed us to form micro-rings with typical diameter of tens of microns, with Q factors between 400 and 600, and a lasing threshold of 110 μJ cm${}^{-2}$ (see Figure 9 right).

Another simple way to obtain multimode lasing, without any fabrication step, is represented by random lasers. In these systems the lasing effects is due to constructive interference of light coherently scattered during the propagation in the active material [76,77].

The first NCs random laser was obtained in CdSe/ZnS core–shell QDs on glass substrates with scratched grooves, showing lasing around 600 nm with a threshold of 25 mJ cm${}^{-2}$ under nanosecond pumping [78].

The first systematic investigation of the random lasing dependence on the NC type and on the scattering centers type was performed on thin films of three different CdSe/CdS core–shell NCs, protected by extra thick shells [79]. The random lasing resulted from scattering from intentionally added gold nanostars or by morphological defects (roughness, cracks and ridges), allowing to obtain lasing thresholds down to 2.5 mJ cm${}^{-2}$ under nanosecond pumping.

**Figure 10:**
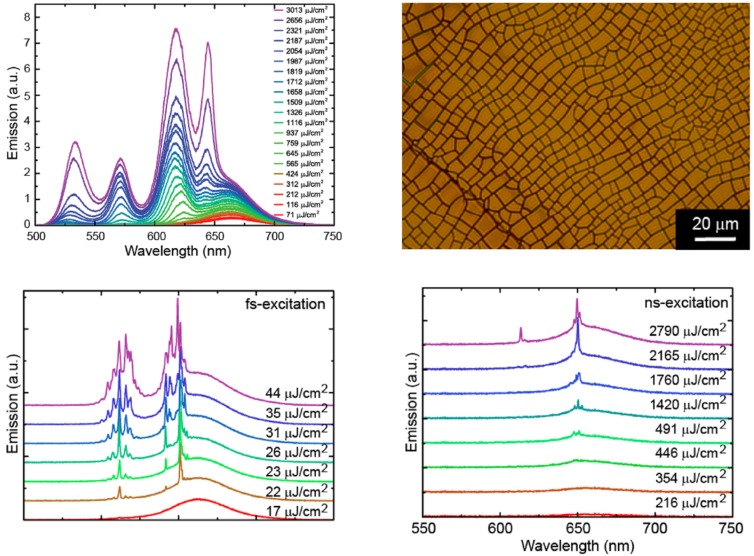
(**top left**) Excitation density dependence of the PL spectra of a blended film of 4.1 and 7.5 nm core diameter CdSe/CdS g-NCs under fs pulses pumping, showing broadband optical gain from 510 to 650 nm. (**top right**) Microscope image of an area with high density of cracks of the film of g-NCs with a 7.5 nm core diameter. (**bottom left**) Emission spectra showing simultaneous random lasing in different bands under femtosecond pulsed excitation. (**bottom right**) Emission spectra showing simultaneous random lasing in different bands under nanosecond pulsed excitation. Adapted from [80]. American Chemical Society, 2016.

The possibility to obtain optical gain from both the core and the shell states in CdSe/CdS giant-shell NCs (g-NCs) recently allowed the demonstration of multiband ASE from two different states of the core and from the shell, and dual band random lasing in which the feedback comes from scattering by cracks on the film surface (see Figure 10), with a threshold strongly decreased to 22 μJ cm${}^{-2}$.

Concerning polymer-NCs hybrid active materials, random lasing has also been demonstrated in CdSe/ZnS core–shell QDS, dispersed in an inhomogeneous polymeric matrix [81]. The system was realized by filling a capillary tube with a mixture of QDs, acrylic resin and NOA65, exploiting the different polymerization rate of two monomers in order to induce local refractive-index fluctuations, thus leading to the formation of spatially localized random resonators. The lasers showed a threshold of 7 mJ cm${}^{-2}$ at 615 nm under 8 ns pump pulses.

Despite their possible realization with simple techniques WGM and random lasers typically generate multimode laser emission with poor directionality. In order to have single mode operation and good laser beam quality more complex and controlled cavity geometries are needed, like distributed feedback (DFB) cavities and vertical cavity surface emitting lasers (VCSELs).

DFB laser resonators have a planar geometry and exploit constructive interference of the light propagating along the film from a periodic grating perpendicular to the propagation direction, that can be obtained when the propagating light wavelength λ satisfies the relation $m\lambda =n_{eff}\Lambda$, where *m* is an integer giving the diffraction order, $n_{eff}$ the effective refractive index and Λ the grating period. The capability to realize periodic grating with a good control of the period allows to combine single mode operation with efficient amplification along the active film.

The first demonstration of DFB lasing from NCs films exploited a CdSe NC/titania film deposited on a DFB grating with a period of 350 nm [82] showing single mode operation with peak wavelength variable from 560 nm to 625 nm by varying both the size of the NCs and the waveguide refractive index. The same group was also the first to realize DFB lasers by soft lithography hot embossing technique, replicating the grating by an elastomeric PDMS mold, and transferring it on the NC/titania film heated at the glass transition temperature [83].

**Figure 11:**
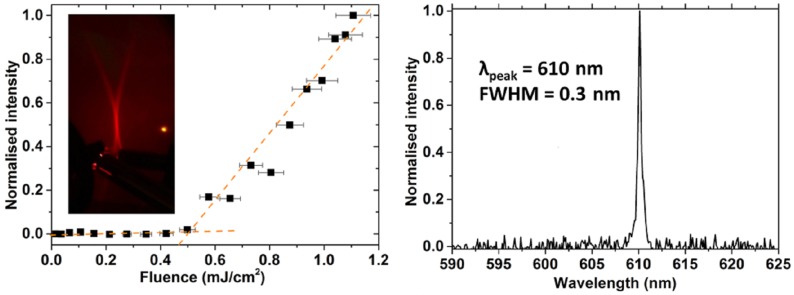
(**left**) Excitation density dependence of the emitted intensity of the CdSe/ZnS-PMMA distributed feedback (DFB) laser, evidencing the presence of a clear lasing threshold. Inset: far field distribution of the DFB emission above threshold. (**right**) Emission spectrum above threshold, evidencing monochromatic and narrow laser emission. Reprinted with permission from [84]. OSA publishing, 2014.

The processing advantages made possible by composite layers realization was recently exploited to realize a DFB laser with a CdSe/ZnS core–shell quantum dots dispersed in a PMMA matrix [84]. The DFB grating was realized on a silica master and then replicated by coating with an optical adhesive in contact with an acetate foil. After the UV curing of the adhesive the master was removed, completing the device by spin coating of the active blend. The high quality of the grating and the good film forming properties allowed by the dispersion in PMMA allowed us to obtain DFB lasing under 5 nanoseconds pumping with narrow with a remarkably low threshold of 0.5 mJ cm${}^{-2}$ (100 kW cm${}^{-2}$) and monochromatic and narrow single mode emission, making them suitable for pumping with compact solid-state lasers (see Figure 11). The same device was also used for demonstrating, for the first time, refractive index sensing by a NCs laser.

Red, green and blue DFB lasing were also demonstrated under 400 ps pumping in close packed films of CdSe/ZnCdS core/shell QDs [85] deposited on a grating realized on a quartz substrate. The lowest threshold of 120 μJ cm${}^{-2}$ was obtained for the red laser, that also evidenced a power conversion efficiency of 28%.

Further improvements in terms of threshold lowering under nanosecond pump pulses were recently obtained [86] in a bilayer DFB lasers with an active alloyed core/shell CdS${}_{x}$Se${}_{1-x}$/ZnS quantum dots deposited on a pre-patterned silica substrate and over-coated with a polyvinyl alcohol (PVA) layer. The PVA layer acts as an encapsulating barrier for oxygen and allows to have a more symmetric refractive index profile, thus improving the mode confinement in the active layer and increasing the gain. The authors demonstrated that the QDs films deposited on planar substrates show ASE with a threshold of 800 μJ cm${}^{-2}$ for the QD film, reduced to 380 μJ cm${}^{-2}$ in the QD/PVA bilayer. The DFB laser showed TE0 emission at 590 nm with a threshold of 85 μJ cm${}^{-2}$ with a QDs active layer, and at 600 nm with a much lower threshold of 13.5 μJ cm${}^{-2}$ with the QD/PVA bilayer.

The last type of resonator that we will investigate is the vertical cavity surface emitting laser (VCSEL). With respect to DFB typically VCSEL are characterized by better beam quality, lower threshold and higher efficiency and, for a proper active layer thickness, can also show single mode operation. However VCSELs cavities are typically more difficult to realize, as a precise control of the active layer thickness is mandatory to properly tune the cavity mode, and good optical quality is necessary to ensure low propagation losses. To the best of our knowledge only three NCs VCSELs have been demonstrated to date.

The first quantum dots based VCSELs were reported by using engineered CdSe/ZnCdS core/shell colloidal pyramid shaped NCs with aromatic ligands, which form densely packed films exhibiting optical gain across the visible spectrum with less than one exciton per colloidal quantum dot on average [87]. The laser cavity was realized by fabricating a wedge films between two dielectric mirrors and allowed us to obtain optically pumped lasing under 100 fs laser pulses in the blue, green and red with ASE threshold of 800 μJ cm${}^{-2}$, 145 μJ cm${}^{-2}$ and 90 μJ cm${}^{-2}$, respectively and VCSEL lasing in the red (615 and 625 nm) with a threshold of 60 μJ cm${}^{-2}$.

More recently the first monolithic VCSEL laser has been demonstrated [88] by using CdSe/CdS/ZnS core/multishell NCs deposited on a bottom DBR mirror and with a top DBR mirror directly deposited on the active layer. The active film showed ASE at 618 nm with a threshold of 6.0 mJ cm${}^{-2}$ under 9 ns pump, while single mode lasing was observed at 623 nm with a threshold of 20 mJ cm${}^{-2}$.

The last example of NCs VCSEL laser has been reported integrating an active dot-in-rod (DiR) CdSe/CdS NCs thin film with two highly reflective polymer dielectric mirrors prepared by spin-coating of alternated layers of polyacrylic acid and poly(N-vinyl carbazol) [89]. The laser showed a main lasing mode at 640 nm with a threshold of about 50 μJ cm${}^{-2}$ under 70 fs pulses, and was proposed as possible initial step for the development of freestanding plastic NCs VCSELs.

The main data about the performances of the described devices are summarized in Table 1.

### 3.2. Light Emitting Diodes

The narrow PL spectrum, the wide emission tunability, the high emission efficiency and the several chemical strategies to engineer the colloidal NCs properties strongly stimulated the research on NCs light emitting diodes. The actual dimension of the performed research is very broad, as evidenced by the more that 5000 papers published on Scopus indexed journals on “NCs” and “LED”, that makes it impossible to cover in a single paper. For this reason in this paper we will focus our attention only on the main developments steps of LEDs exploiting organic–inorganic blends as active layers. Many other specific aspects of QDs LEDs are already covered by other excellent reviews about the device structure, working principle and commercial applications perspectives [24,25,26,27,28].

The combination of organic and NCs for the realization of NC-LEDs was actually introduced already in the first report on QDs electroluminescence [90]. The devices exploited an active multilayer made by alternated CdSe QDs layers, and PPV organic layers, and showed a minimum turn on voltage of about 4 V, a luminance up to about 100 cd m${}^{-2}$, tunable electroluminescence from red to yellow by changing the QDs size and voltage dependent emission color, due to the simultaneous excitation of both PPV and CdSe electroluminescence.

The first device exploiting a polymer-NCs blend was reported one year later [91]. The active layer was a blend between CdSe QDs and a mixture of a hole transporting polyvinyl-carbazole (PVK), and an electron transporting oxadiazole derivative (t-Bu-PBD). In this case the electroluminescence spectra of the devices only showed the contribution of CdSe, resulting in a narrow emission band (FWHM about 40 nm) around 620 nm. The external quantum efficiency (EQE) was estimated in 0.0005%, clearly evidencing the need of device optimization.

The combination of efficient FRET energy transfer from the polymer to the NC and the good quality of the used core/shell NCs allowed the demonstration of efficient monochromatic emission at 630 nm with luminance efficiency up to 0.32 cd/A [92] in LEDs exploiting poly(2,7-spirofluorene)(PSF) and red-emitting CdSe/ZnS core–shell QDS blends.

The importance of engineering the energy transfer between the polymer and the NCs and the phase segregation between the components has been demonstrated by developing functionalized polymers in order to reduce the NCs segregation, allowing an increase of the EQE of one order of magnitude in nanocomposites with respect to blends [93].

**Figure 12:**
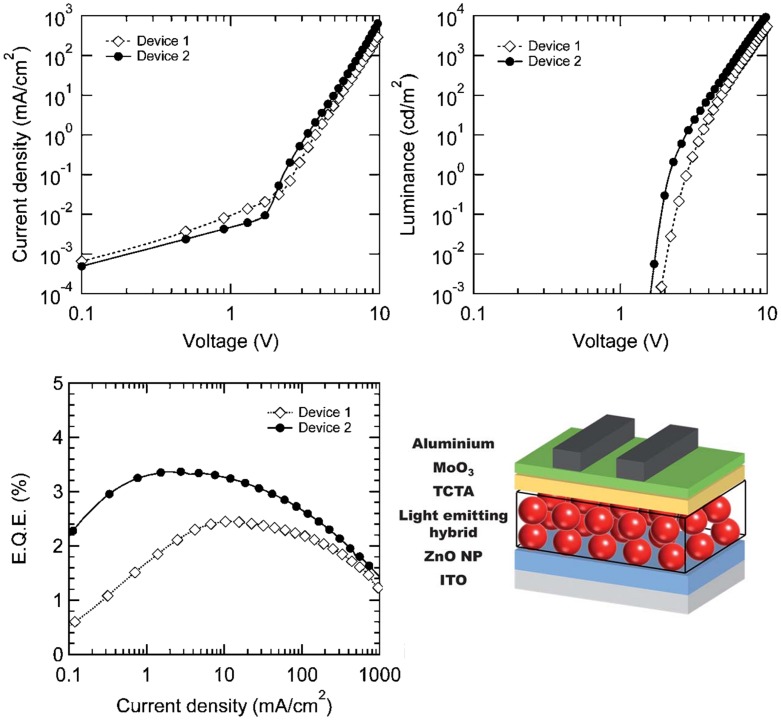
Voltage dependence of the current density (**top left**), luminance (**top right**) EQE (**bottom left**) and device structure of the light emitting diode (LED) realized in [94]. Adapted with permission from [94]. The Royal Society of Chemistry, 2013.

Moving to more recent devices the combination of optimized hole injecting electrode and polymer-NCs hybridization thanks to properly functionalized polymers recently allowed us to obtain a high EQE of 3.37% in LEDs with CdSe/CdS/CdZnS red QDs-polymer blends [94] (see Figure 12).

A particularly interesting consequence of the possibility to realize hybrid layers generating light with the contribution both of the organic and of the NCs is the generation of white light emitting systems.

The first white hybrid LED exploited a blend between a blue emitting polymer (PDHFPPV) and CdSe QDs of two different sizes in order to have green and red emission, carefully controlling the blend composition in order to prevent complete energy transfer from the polymer to the QDs [95].

White light generation was also observed in LEDs with simpler binary polymer-NCs blends. In particular a combination of PVK and CdSe/CdS core/shell QDs was used [96] to obtain white emission with a turn on voltage of 10 V, a luminance of 180 cd m${}^{-2}$ and a maximum current efficiency of 0.21 cd A${}^{-1}$ A strong efficiency increase up to 0.24% was obtained by combining a first layer made by a blend between a polyfluorene derivative (PFH-MEH) and CdSe/ZnS QDs with a top Alq3 layer, that improves the charge injection properties, and improves the white color balancing [97].

Another experiment on binary organic–inorganic blends was performed combining a blue–green emitting organic material (BADF) with CdSe/ZnS core/shell QDs [98], evidencing as critical issues a voltage dependent spectrum, and a LED performances decrease, due to an efficiency reduction and a turn on voltage increase, caused by the NCs presence in the blend, ascribed to minority charge carrier trapping from the NCs.

Moving to more recent experiments a quaternary active layer made by a commercial blue emitting polymer (ADS329BE) blended with blue CdZnS/ZnS, green CdSe/ZnS core/shell, and red CdSe/CdS/ZnS core/shell/shell QDs [99] was recently realized. The optimized active material and device structure allowed us to strongly improve the performances leading to low turn on voltage, high current efficiency and luminance.

The main performances of the discussed devices are summarized in Table 2.

## 4. Perspectives

As evident from the previously described results the wide research on organic molecule and NCs allowed strong improvements of the understanding of the emission properties of hybrid organic–inorganic systems, and of the laser and LEDs based on these materials. However, to date no real devices based on these materials are present on the market, evidencing the need of further advances to get this result.

Concerning lasers, the possible next steps could involve the development of optically pumped devices with a low cost pumping source, for example pulsed LEDs, already successfully employed to pump organic lasers [100] and the extension of systems showing CW lasing.

Moreover, the actual state of the art of organic–inorganic based lasers only include hybrid combination of inert polymers with active NCs, while active host-guest blends are widely used in the fully organic laser community to reach low threshold lasing. Assuming a similar effect of the dispersion in an active matrix also for NCs based lasers, improvements in terms of threshold lowering could be expected.

Concerning LEDs, to date the performances of devices exploiting hybrid active materials are still lower than the ones of inorganic NCs LEDs. This situation evidences that to date the chemical approaches to allow good film uniformity and high charge injection and transport in NCs thin films outperformed the attempts to exploit active organic material to improve these aspects. In this case however a very active and wide research is still ongoing and the possibility to exploit further degrees of freedom related to the engineering possibility of the organic component and of its combination with the inorganic part is expected to allow other performances improvements in the future.

**Figure 13:**
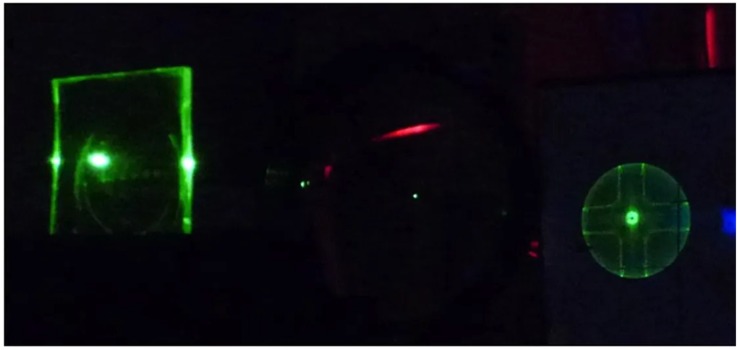
Photograph of a MAPbBr${}_{3}$ DFB laser above the lasing threshold. Adapted with permission from [102]. Springer Nature Publishing, 2017.

Among the possible developments of organic–inorganic materials containing semiconductor NCs the recent breakthrough of demonstration of perovskites NCs synthesis [32] is expected to allow the investigation of new physics and to further improve the performances both of lasers and LEDs. Even if these nanocrystals have different chemical properties with respect to the II–VI nanocrystals discussed in this paper, they have in common with II–VI nanocrystals the wide chemical flexibility of the synthesis in solution, the physical active properties and the possible complementarity with the polymer processing and physical properties. For these reasons polymer–perovskite NCs blends are expected to provide interesting templates for future photophysics experiments and for further applicative advances. In particular it is relevant to underline that perovskites already demonstrated an impressively rapid improvement of lasing properties that lead from the first demonstration of optical gain under femtosecond optical pumping in NCs [101], to ASE and lasing demonstration under nanosecond pulsed pumping [102,103,104] (see Figure 13), to the first evidence of ASE under CW pumping in only 3 years [105]. In a similar way perovskites LEDs currently reached an EQE of 17.5%, with luminance up to 5 $\times 10^{4}$ lmW${}^{-1}$ an turn on voltage around 3 V [106] in only 5 years from the first electroluminescence evidence [107], while perovskites NCs demonstated fully tunable and narrow emission, covering up to 140% of the NTSC color standard [32] (see Figure 1 bottom right). Hybrid organic–inorganic materials including perovskites NCs have been also already demonstrated both for the realization of color conversion layers by dispersion in inert polymer and also in active polymer-NCs blends, exploiting the superposition of the luminescence of the two component to generate white light [108], thus proposing these systems as candidates for white LEDs for display and lighting applications.

## Figures and Tables

**Table 1:** Amplified Spontaneous Emission and lasing properties of light amplifying active materials based on nanocrystals (NCs) and polyer-NCs hybrids. The data are ordered taking into account the cavity type and in order of increasing pump pulse time width.

Active Material	Cavity Geometry	Pump Pulse Length	Threshold	Peak Wavelength (nm)	Ref.
CdSe/CdS g-NCs	ASE	70 fs	45–105 μJ cm${}^{-2}$	612, 648 (dual band)	[80]
CdSe/CdS DiR	ASE	70 fs	115 μJ cm${}^{-2}$	628	[71]
CdSe/Zn${}_{0.5}$Cd${}_{0.5}$S pyramids	ASE	100 fs	800 μJ cm${}^{-2}$	450	[87]
CdSe/Zn${}_{0.5}$Cd${}_{0.5}$S pyramids	ASE	100 fs	145 μJ cm${}^{-2}$	565	[87]
CdSe/Zn${}_{0.5}$Cd${}_{0.5}$S pyramids	ASE	100 fs	90 μJ cm${}^{-2}$	625	[87]
CdZnS QDs	ASE	100 fs	260 μJ cm${}^{-2}$	472	[63]
CdZnS/ZnS QDs SS	ASE	100 fs	160 μJ cm${}^{-2}$	468	[63]
CdZnS/ZnS QDs AS	ASE	100 fs	55 μJ cm${}^{-2}$	463	[63]
CdSe/ZnS QDs-PMMA	ASE	5 ns	1.0 mJ cm${}^{-2}$	611	[84]
CdS${}_{x}$Se${}_{1-x}$/ZnS	ASE	5 ns	800 μJ cm${}^{-2}$	600	[86]
CdS${}_{x}$Se${}_{1-x}$/ZnS-PVA	ASE	5 ns	380 μJ cm${}^{-2}$	600	[86]
CdSe/CdZnS QDs-titania	microsphere	100 fs	8.1 mJ cm${}^{-2}$	∼660 nm	[68]
CdSe/CdS DiR	microring	70 fs	15 μJ cm${}^{-2}$	632	[71]
CdSe/CdS DiR	microring	100 fs	200 μJ cm${}^{-2}$	495.5	[70]
CdSe/CdS QDs	microring	100 fs	2 μJ cm${}^{-2}$	636	[72]
CdSe/CdS QRs	microring	150 fs	3.0 mJ cm${}^{-2}$	∼660 nm	[69]
CdSe/CdS/ZnS QDs	microring	340 fs	22 μJ cm${}^{-2}$	610	[73]
CdSe/CdS/ZnS QDs	microring	340 fs	110 μJ cm${}^{-2}$	530	[73]
CdSe/ZnS QDs-PVP	microring	300 ps	110 μJ cm${}^{-2}$	600	[75]
CdSe/CdS g-NCs	random	70 fs	22 μJ cm${}^{-2}$	610, 650 (dual band)	[80]
CdSe/CdS QDs	random	1.3 ns	2.5 mJ cm${}^{-2}$	610 nm	[79]
CdSe/CdS g-NCs	random	3ns	490 μJ cm${}^{-2}$	610, 650 (dual band)	[80]
CdSe/ZnS QDs	random	5 ns	25 mJ cm${}^{-2}$	600 nm	[78]
CdSe/ZnS QDS-NOA65-resin	random	8 ns	7 mJ cm${}^{-2}$	615 nm	[81]
CdSe/ZnCdS CQDs	DFB	400 ps	330 μJ cm${}^{-2}$	455 nm	[85]
CdSe/ZnCdS CQDs	DFB	400 ps	280 μJ cm${}^{-2}$	575 nm	[85]
CdSe/ZnCdS CQDs	DFB	400 ps	120 μJ cm${}^{-2}$	610 nm	[85]
CdSe/ZnS QDs-PMMA	DFB	5 ns	0.5 mJ cm${}^{-2}$	610 nm	[84]
CdS${}_{x}$Se${}_{1-x}$/ZnS	DFB	5 ns	85 μJ cm${}^{-2}$	590 nm	[86]
CdS${}_{x}$Se${}_{1-x}$/ZnS-PVA	DFB	5 ns	13.5 μJ cm${}^{-2}$	600 nm	[86]
CdSe/CdS DiR	VCSEL	70 fs	50 μJ cm${}^{-2}$	640 nm	[89]
CdSe/Zn${}_{0.5}$Cd${}_{0.5}$S	VCSEL	100 fs	60 μJ cm${}^{-2}$	615, 625	[87]
CdSe/CdS/ZnS	VCSEL	9 ns	20 mJ cm${}^{-2}$	623 nm	[88]

**Table 2:** Performances of the hybrid light emitting diodes (LEDs) described in the text.

Active Layer	EL Peak	EL FWHM	EQE	Curr. Eff.	Pow. Eff.	Lumin.	Turn on Volt.	Ref.
	(nm)	(nm)	%	cdA${}^{-1}$	lmW${}^{-1}$	cdm${}^{-2}$	(V)	
CdSe QDs-PPV multilayer	about 600	N/A	0.001–0.01	N/A	N/A	100	4	[90]
CdSe QDs-PVK-(t-Bu-PBD)	620	40	0.0005	N/A	N/A	N/A	N/A	[91]
CdSe/ZnS QDs-PSF	630	N/A	N/A	0.32	N/A	30	7	[92]
CdSe/CdS/CdZnS QDs-BCP	630	30	3.37	2.71	1.26	10000	2	[94]
CdSe/CdS QDs-PVK	white	N/A	N/A	0.21	N/A	180	10	[96]
(PFH-MEH)-CdSe/ZnS QDs	white	N/A	0.24	N/A	N/A	N/A	9	[97]
CdSe/ZnS QDs-BADF	white	N/A	0.02	0.054	0.017	N/A	6.5	[98]
RGB QDs-ADS329BE	white	N/A	N/A	1.43	N/A	15950	1.5	[99]
